# Effects of anti‐IL5 biological treatments on blood IgE levels in severe asthmatic patients: A real‐life multicentre study (BIONIGE)

**DOI:** 10.1002/clt2.12143

**Published:** 2022-04-07

**Authors:** Marco Contoli, Pierachille Santus, Francesco Menzella, Cindy Rocchi, Dejan Radovanovic, Federico Baraldi, Chiara Martelli, Serena Casanova, Carlo Barbetta, Claudio Micheletto, Nicola Scichilone, Bianca Beghè, Elisiana Carpagnano, Alberto Papi

**Affiliations:** ^1^ Respiratory Medicine Department of Translational Medicine University of Ferrara Ferrara Italy; ^2^ Emergency Department University Hospital S. Anna Ferrara Italy; ^3^ Division of Respiratory Diseases, Ospedale Luigi Sacco Polo Universitario, ASST Fatebenefratelli‐Sacco Department of Biomedical and Clinical Sciences (DIBIC) Università Degli Studi di Milano Milan Italy; ^4^ Pneumology Unit Arcispedale Santa Maria Nuova, Azienda USL‐IRCCS di Reggio Emilia Reggio Emilia Italy; ^5^ Department of Pulmonary Medicine Ospedale Santa Maria degli Angeli Pordenone Italy; ^6^ Cardio‐Thoracic Department, Respiratory Unit Integrated University Hospital Verona Italy; ^7^ Dipartimento Universitario di Promozione Della Salute, Materno Infantile, Medicina Interna e Specialistica di Eccellenza "G. D'Alessandro" (PROMISE) Division of Respiratory Medicine "Paolo Giaccone" University Hospital University of Palermo Palermo Italy; ^8^ Respiratory Diseases Unit Department of Medical and Surgical Sciences University of Modena Reggio Emilia Italy; ^9^ Division of Respiratory Diseases Department of Medical and Surgical Sciences Respiratory and Critical Care Unit University of Foggia Polyclinic University Hospital Bari Italy

**Keywords:** basophils, benralizumab, eosinophils, Ig‐E, mepolizumab, severe asthma

## Abstract

**Background:**

Mepolizumab and benralizumab are clinically effective biological treatments for severe eosinophilic asthmatic patients by hampering eosinophilic inflammation. The effects of these compound on the immunoglobulin (Ig)E T2 component are virtually unknown.

**Objectives:**

To evaluate the change in total IgE levels at 4 ± 2 months after initiation of the mepolizumab (primary outcome) or benralizumab. When available, the changes of blood inflammatory cell counts, lung function and asthma control test (ACT) were also assessed and correlated with changes in total IgE levels.

**Methods:**

Observational, retrospective, multicentre, cohort study. Severe eosinophilic atopic asthmatic patients treated with mepolizumab or benralizumab were included in the analysis.

**Results:**

Three‐month treatment (on average) with mepolizumab (*n* = 104) or benralizumab (*n* = 82) resulted in significantly higher reduction of blood eosinophil and basophil levels in patients treated with benralizumab compared to mepolizumab. Mepolizumab did not significantly modified the levels of blood total IgE during the study period, whereas benralizumab significantly reduced (−35%, *p* < 0.001) total blood IgE levels. In patients treated with benralizumab the reduction of blood total Ig‐E levels correlated with the reduction of blood basophils (but not eosinophils) and weakly with the improvement of asthma control.

**Conclusion:**

Benralizumab but not mepolizumab, treatment led to a significant reduction of circulating IgE level. The study provides different and specific mechanisms of action for anti‐IL5‐pathway treatments.

## 1 INTRODUCTION

The management of severe asthma is a major unmet medical need in respiratory medicine.^1^ Severe asthmatic patients are not clinically controlled despite optimised treatment or require maximal treatment to achieve asthma control.^2^, ^3^ Major advances have been made in the past few years in the management of severe asthma given the development of targeted biological therapies.^4^, ^5^

Mepolizumab and benralizumab are humanised monoclonal antibodies directed against interleukin (IL)‐5 and a subunit of the IL‐5 receptor (IL‐5R), respectively.^6^, ^7^ They inhibit eosinophil‐driven inflammation by blocking IL‐5 or its receptor.^6^, ^7^ Clinically, these compounds can improve the quality of life, reduce the need for oral corticosteroid use, and reduce the exacerbation rate in patients with severe asthma with elevated blood eosinophil counts.^8^, ^9^, ^10^, ^11^, ^12^, ^13^

Anti‐IL‐5 and anti‐IL‐5R monoclonal antibodies share several mechanisms of action; however, targeting IL‐5 or its cellular receptor (IL‐5R) can interfere with different inflammatory pathways.^6^, ^7^ Understanding the specific mechanisms of action of biological treatments can help clinicians select the most appropriate treatment for the right patient.

The effects of these differences on other key effector and regulatory molecules of the T2‐immune response, such as immunoglobulin (Ig) E, are virtually unknown. IgE is a pleiotropic molecule that acts as a key inflammatory molecule in the T2‐inflammatory cascade as well as a modulatory molecule of innate and adaptive immune systems.^14^ Augmentation of the total IgE levels is common in asthma irrespective of the underlying atopic status.^15^ Targeting blood circulating IgE has proven effective in improving clinical outcomes under different conditions characterised by high levels of blood IgE, including severe atopic asthma.^16^, ^17^, ^18^ Thus, it is worth investigating whether anti‐IL‐5 or anti‐IL‐5R treatments impact blood IgE levels.

To investigate the effects of treatments interfering with the IL‐5 pathways on IgE levels, we performed a multicentre study primarily aimed at evaluating the effect of anti‐IL‐5 mepolizumab on circulating total IgE levels. To assess the specificity of the effect, a cohort of patients treated with the anti‐IL‐5 receptor monoclonal antibody benralizumab was also evaluated.

## 2 METHODS

### 2.1 Study population

Patients with severe eosinophilic asthma [according to the Global Initiative for Asthma (GINA) document^2^] treated with mepolizumab or benralizumab were included in the study. According to the Italian Medicines Agency (AIFA), the indications required for mepolizumab prescription were >150 blood eosinophils/mcl while not receiving systemic corticosteroids or >300 blood eosinophils/mcl in the previous year, and at least one of the following conditions: (i) two or more severe exacerbations requiring systemic corticosteroids in previous years despite maximised inhaled treatment (GINA treatment steps 4 and 5); (ii) requirement of systemic corticosteroids of top of inheld treatment for at least 6 months in the previous year. The indications for benralizumab were as follows: >300 blood eosinophils/mcl while not receiving systemic corticosteroids, and at least one of the following conditions: (i) two or more severe exacerbations requiring systemic corticosteroids in the previous years despite maximised inhaled treatment (GINA treatment steps 4 and 5); (ii) requirement of systemic corticosteroids of top of inhaled treatments.

The analyses were conducted in patients with severe asthma receiving mepolizumab or benralizumab, for which the following information was available: (i) positive atopic status. Atopy was defined as a positive detection of specific IgE (RAST test) to any allergen (including house dust mites, dogs, cats, and seasonal pollen, such as grasses, birch, cypress, hazel, Alder, and *Alternaria*); (ii) total IgE levels in stable conditions within 6 months prior to the beginning of the anti‐IL5/Ra treatments and a second assessment at 4 ± 2 months after the initiation of the biologic treatments. Total IgE values should be obtained under stable conditions, that is, free from any exacerbations for at least 8 weeks. Within 6 months before the initiation of treatment with biologics, IgE assessment closer to the date of treatment initiation was considered. The first IgE assessment was performed 2–6 months from the date of initiation of treatment with biologics. Only patients with repeated measurements of blood IgE levels within the study timeframe were screened and included in the analysis. Measurements of blood inflammatory cell counts, fractional nitric oxide (NO) concentration in exhaled breath (FeNO), and clinical outcomes were also considered if performed within the same timeframes and with the same temporal criteria. Patients receiving chronic oral corticosteroids (defined as therapy for at least 6 continuous months before enrolment^13^) were excluded from the analysis.

### 2.2 Study design

This is a real‐life (Phase IV), observational, retrospective, multicentre, and cohort study. Eight centres participated in the study. All centres had dedicated clinics for severe asthma. A list of centres is provided in Appendix. The study conformed to the Declaration of Helsinki and was approved by the institutional ethics committee. Informed written consent was obtained from all participants. The study was registered at ClinicalTrials.gov (identifier NCT04181190).

### 2.3 Aims and measurements

The study was designed to primarily evaluate the changes in total IgE levels (kU/l) in atopic patients with severe eosinophilic asthma at 4 ± 2 months after initiation of the anti‐IL‐5 mepolizumab treatment. To assess the specificity of the intervention, we also investigated the changes in total IgE levels (kU/l) in a similar population of atopic patients with severe eosinophilic asthma at 4 ± 2 months after initiation of the anti‐IL5R monoclonal antibody benralizumab treatment. We further assessed blood inflammatory cell counts before monoclonal antibody treatment and 4 ± 2 months after the initiation of each of the two treatments. When available during the study timeframe, lung function, fractional nitric oxide (NO) concentration in exhaled breath (FeNO) and asthma control test (ACT) measurements^19^ at baseline before initiation of monoclonal antibody treatments and at 4 ± 2 months after initiation of treatment were also collected for each of the two biologics.

### 2.4 Statistical analysis

A formal calculation of the sample size for this study was not possible, as no clinical study has specifically assessed the effect of anti‐IL‐5 monoclonal antibodies on circulating total IgE levels in patients with severe eosinophilic allergic asthma (the primary outcome of this study). Therefore, we estimated the sample size considering (a) mean values of total baseline IgE described in a population of patients with severe asthma^20^ and (b) an expected 35% reduction in the total IgE level that has been reported with other biologic treatments.^21^ Based on these assumptions, we calculated that 104 patients on mepolizumab therapy would be sufficient to address the primary outcome of this study (alpha 0.05, power 0.8). Assessment of clinical outcomes of mepolizumab and benralizumab treatments only had a descriptive value; the comparison of effectiveness between treatments was beyond the statistical power of the present study.

Data are shown as the mean ± SD unless otherwise specified. The standardised difference between means (for continuous variables) and between prevalence (for dichotomous variables) was calculated to compare the baseline characteristics of the mepolizumab‐ and benralizumab‐treated groups.^22^, ^23^ The changes in biomarkers and inflammatory cell counts were assessed using the paired *t*‐test or Wilcoxon test, as appropriate. Comparisons between groups were assessed using the unpaired *t*‐test or Mann‐Whitney *U* test, as appropriate. Correlation coefficients were calculated using the Spearman's rank method. *p*‐values of 0.05 or less were considered to indicate statistical significance. Post hoc Holm‐Bonferroni correction was applied for multiple testing.

## 3 RESULTS

### 3.1 Study population

The medical records of 165 patients with severe eosinophilic asthma treated with mepolizumab with repeated measurements within the study timeframe of blood IgE levels were screened to obtain a cohort of 104 patients fulfilling the inclusion criteria of the analysis. A total of 28 patients were excluded from the analysis because they were non‐atopic. Another 33 patients (10 non‐atopic and 23 atopic patients) were excluded because of chronic oral corticosteroid treatment. Eighty‐two atopic severe eosinophilic asthmatic patients treated with benralizumab who fulfilled the inclusion/exclusion criteria were also included in the specificity analysis. The patient characteristics are shown in Table 1 and in the online supportive information. No statistically significant differences were found between the two groups at baseline before the biological treatments. To further evaluate the match between study groups, standardised differences in baseline clinical, functional, and biological variables were calculated (Table S1 in Supporting Information S1). Standardised differences between groups were laid within a 10% window (standardised difference <0.1) that met the criterion of a negligible difference.^22^, ^23^ Further information is available in Supporting Information S1. The durations of biologic treatments before assessing the IgE change were similar in the two groups of patients (117 ± 4 days and 108 ± 4 days for mepolizumab and benralizumab, respectively, *p* > 0.05). No major side effects were documented in patients treated with mepolizumab or benralizumab (see also Supporting Information S1).

**TABLE 1:** Study population

	Mepolizumab group (*N* = 104)	*N* (%)	Benralizumab group (*N* = 82)	*N* (%)	*p*
Age, years	57 (12)	104 (100%)	56 (14)	82 (100%)	*p* > 0.05
Female, *n* (%)	55 (53%)	104 (100%)	42 (51%)	82 (100%)	*p* > 0.05
Smoking habit		104 (100%)		82 (100%)	
Current, *n* (%)	8 (8%)		3 (4%)		*p* > 0.05
Former, *n* (%)	29 (28%)		19 (23%)		*p* > 0.05
Pack‐year	11.9 (9.6)		12.4 (9.4)		*p* > 0.05
Lung function		95 (91%)		69 (84%)	
FEV1, litre	1.92 (0.08)		1.95 (0.09)		*p* > 0.05
FEV1, % predicted	70.9 (21.8)		71.7 (22.5)		*p* > 0.05
ACT score	16.4 (4.1)	89 (86%)	15.7 (3.7)	66 (79%)	
Comorbid conditions		104 (100%)		82 (100%)	
Obesity BMI > 30, *n* (%)	18 (17.3%)		11 (13.4%)		*p* > 0.05
Atopic dermatitis, *n* (%)	12 (11.5%)		6 (7.1%)		*p* > 0.05
Chronic rhinosinusitis including nasal polyps, *n* (%)	52 (50%)		43 (51.1%)		*p* > 0.05
Gastroesophageal reflux disease, *n* (%)	23 (22%)		19 (19%)		*p* > 0.05
Anxiety‐depression syndrome, *n* (%)	10 (9.6%)		8 (9.7%)		*p* > 0.05
Total blood IgE, kU/l	404 (476)	104 (100%)	384 (519)	82 (100%)	
Inflammatory cell counts, cells/μl		98 (94%)		76 (93%)	
Neutrophils	5007 (1568)		4770 (1792)		*p* > 0.05
Lymphocytes	2070 (655)		1994 (693)		*p* > 0.05
Basophils	48 (32)		55 (43)		*p* > 0.05
Eosinophils	696 (458)		647 (365)		*p* > 0.05

*Note*: Demographic and clinical characteristics refer to the assessment performed within 6 months from the beginning of biological treatments. Data are presented as mean (SD) unless otherwise indicated (ACT: asthma control test score; BMI: body mass index; FEV1: forced expiratory volume in the 1st second).

### 3.2 IgE levels

**FIGURE 1:**
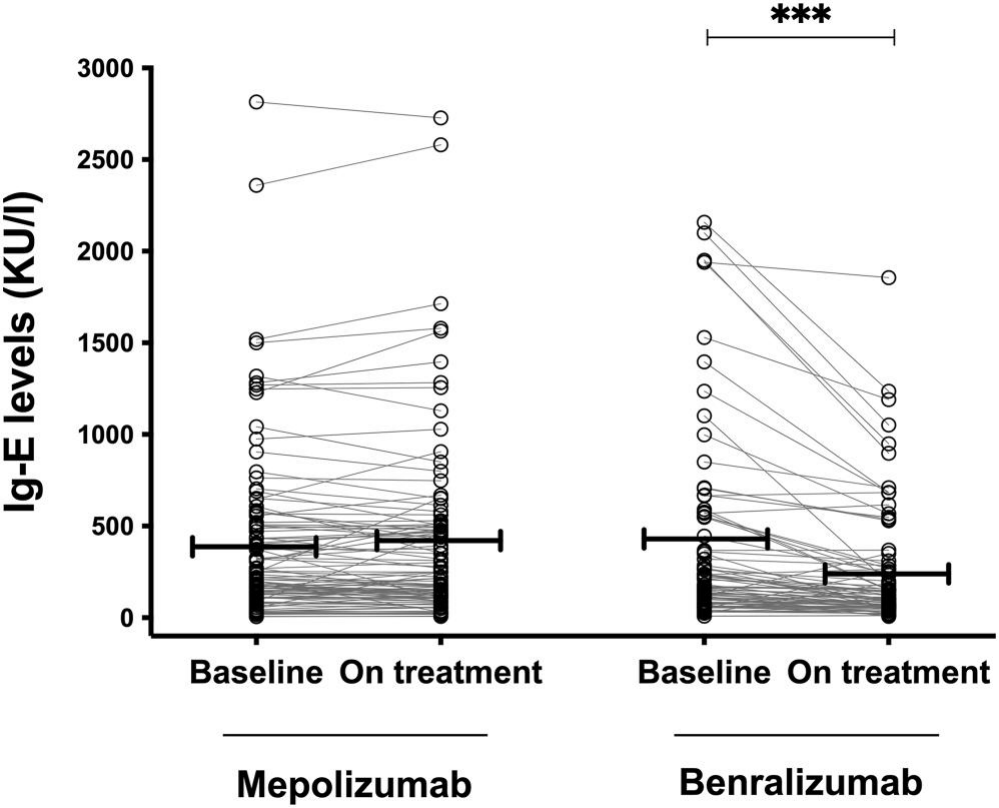
Blood total IgE levels at baseline (before biologic treatments) and on‐treatment in severe eosinophilic asthmatic patients treated with mepolizumab or benralizumab

The measurements of total IgE levels were available for all patients included in the study both at baseline (within 6 months from the beginning of biological treatments) and at 4 ± 2 months after initiation of treatment. Similar levels of total IgE were found in the two groups of patients at baseline before treatment with monoclonal antibodies (Table 1 and Figure 1). This assessment was performed 64 ± 4 and 70 ± 4 days before the initiation of mepolizumab and benralizumab, respectively (*p* > 0.05). Mepolizumab administration did not modify IgE values when assessed after 116 ± 3 days of treatment (387 ± 56 and 404 ± 47 kU/I at baseline and after 116 ± 3 days of treatment with mepolizumab, respectively; Figure 1). Treatment with benralizumab was associated with a significant reduction in total IgE levels compared to baseline (384 ± 57 and 247 ± 35 kU/l at baseline and after 108 ± 4 days of treatment with benralizumab, respectively; −36%, *p* < 0.001; Figure 1). Such a reduction was consistent and independent of the pharmacological regimen adopted (monthly vs. bimonthly administration) and was not biased by an early assessment of IgE levels after administration of benralizumab (detailed in Supporting Information S1). Additional analyses were performed in patients with all demographic, clinical, functional, and biological characteristics available at baseline, and also with the exclusion of patients with low blood eosinophils (<300 cells/mcl) in the mepolizumab treated group (detailed in Supporting Information S1). These analyses provided results similar to those obtained when considering the entire population (detailed in Supporting Information S1).

In mepolizumab‐treated patients (in whom overall, a non‐significant 2% increase in total IgE levels was found compared to baseline), an increase in blood IgE levels was found in 28 patients (27%), a stability in 38 patients (37%), and a reduction in 36 patients (35%). Conversely, a reduction in blood IgE levels was found in the vast majority (92%) of patients treated with benralizumab, with only eight patients showing an increase of at least 35% compared to baseline.

### 3.3 Blood inflammatory cell counts and FeNO measurements

**FIGURE 2:**
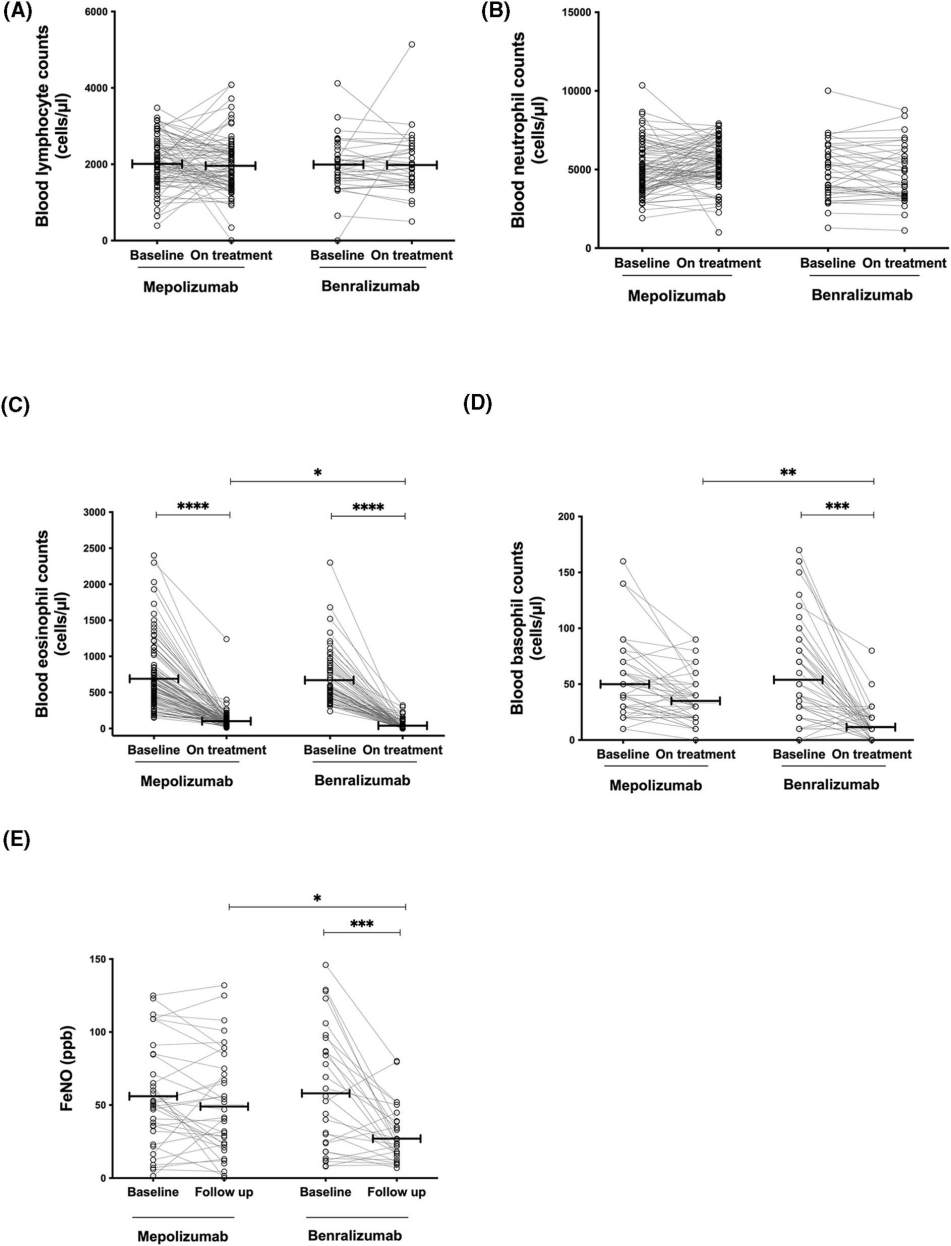
(A–D) Blood inflammatory cell counts at baseline (before biologic treatments) and on‐treatment in severe eosinophilic asthmatic patients treated with Mepolizumab or Benralizumab. (E) FeNo levels at baseline (before biologic treatments) and on‐treatment in severe eosinophilic asthmatic patients treated with mepolizumab or benralizumab

Blood sample tests performed before and after initiation of monoclonal antibody treatments were available for 94% (*n* = 98) of patients treated with mepolizumab and for 88% (*n* = 72) of patients treated with benralizumab. Similar levels of total blood leukocytes, blood lymphocytes, blood eosinophils, and blood basophils were found at baseline in the two patient groups (Table 1). No treatment effect was observed on blood lymphocyte (Figure 2A) and neutrophil (Figure 2B) levels. Both treatments resulted in significant reductions in blood eosinophil counts (Figure 2C). On‐treatment blood eosinophil levels were significantly lower in patients receiving benralizumab than in those receiving mepolizumab (*p* < 0.05; Figure 2C).

Interestingly, we found that benralizumab strongly reduced blood basophil counts, while mepolizumab showed a limited effect (78% vs. 33% reduction, *p* < 0.01, in benralizumab and mepolizumab after 108 ± 3 and 115 ± 3 days, respectively; Figure 2D).

Paired before and after measurements of FeNO were available for more than one‐third of the patients [36 patients (35%) treated with mepolizumab and 28 patients (34%) treated with benralizumab]. In line with a recent publication,^24^ we found that benralizumab but not mepolizumab treatment resulted in a significantly reduction in FeNO levels (*p* < 0.05, Figure 2E).

### 3.4 Correlations between IgE levels and blood inflammatory cell counts

**FIGURE 3:**
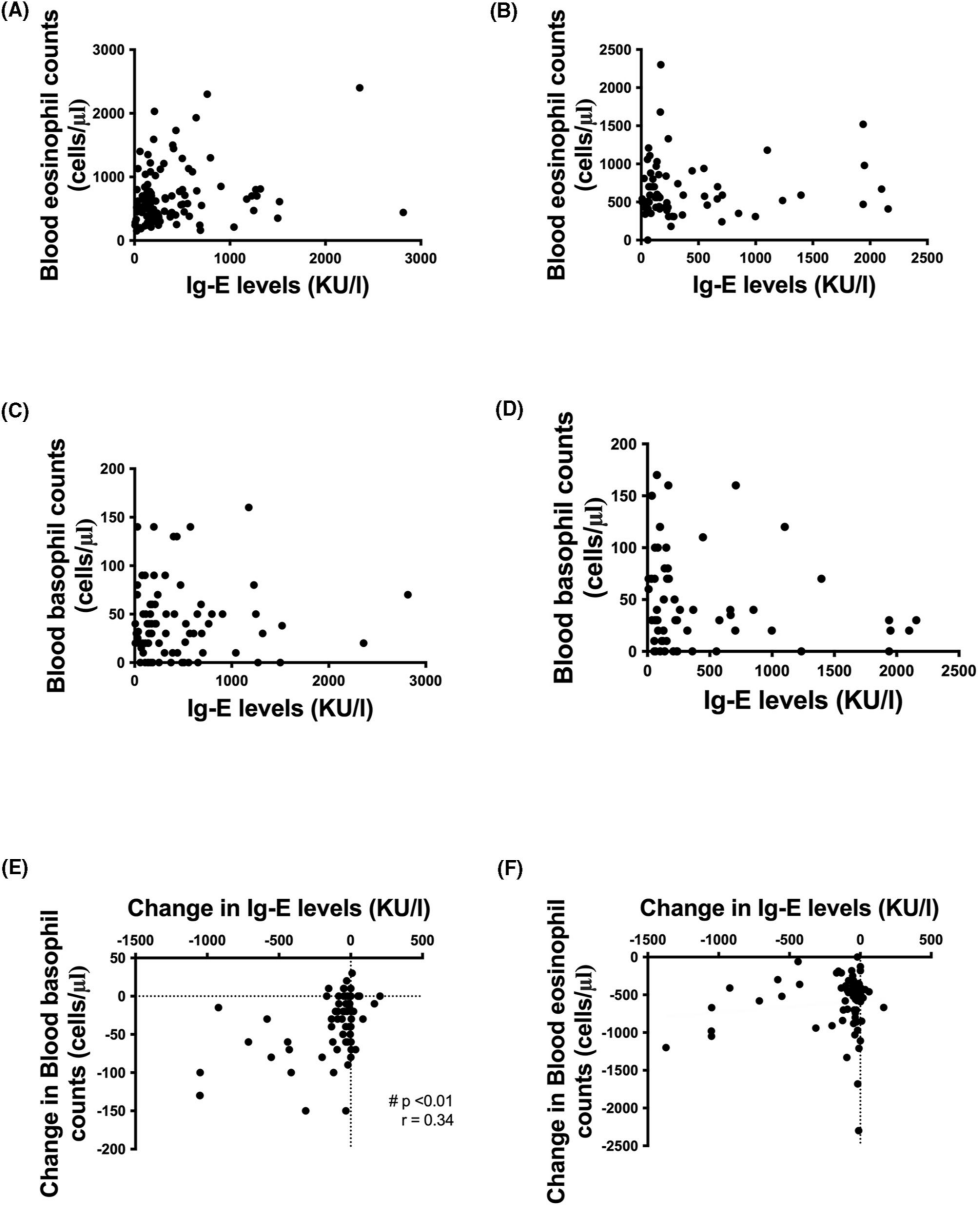
Correlations between IgE levels and blood inflammatory cell counts at baseline (before biologic treatments) in severe eosinophilic asthmatic patients treated with mepolizumab (A and C) or benralizumab (B and D). Correlations between the change in total blood IgE levels and blood basophil (E) or eosinophil (F) counts in patients treated with benralizumab (# unadjusted *p*‐value for multiple testing)

No correlations were found in both groups of patients between IgE levels and blood eosinophil (Figure 3A,B) or basophil (Figure 3C,D) counts, both at baseline (Figure 3A–D) and on treatment (data not shown).

A significant positive correlation was found in patients treated with benralizumab (but not in patients treated with mepolizumab) between the on‐treatment reduction of IgE levels and the reduction of blood basophil levels (*p* < 0.01; *r* = 0.34; Figure 3E) but not of eosinophil counts (Figure 3F). This was confirmed by Holm‐Bonferroni correction for multiple comparisons (adjusted *p*‐value 0.016).

### 3.5 Clinical outcomes

**FIGURE 4:**
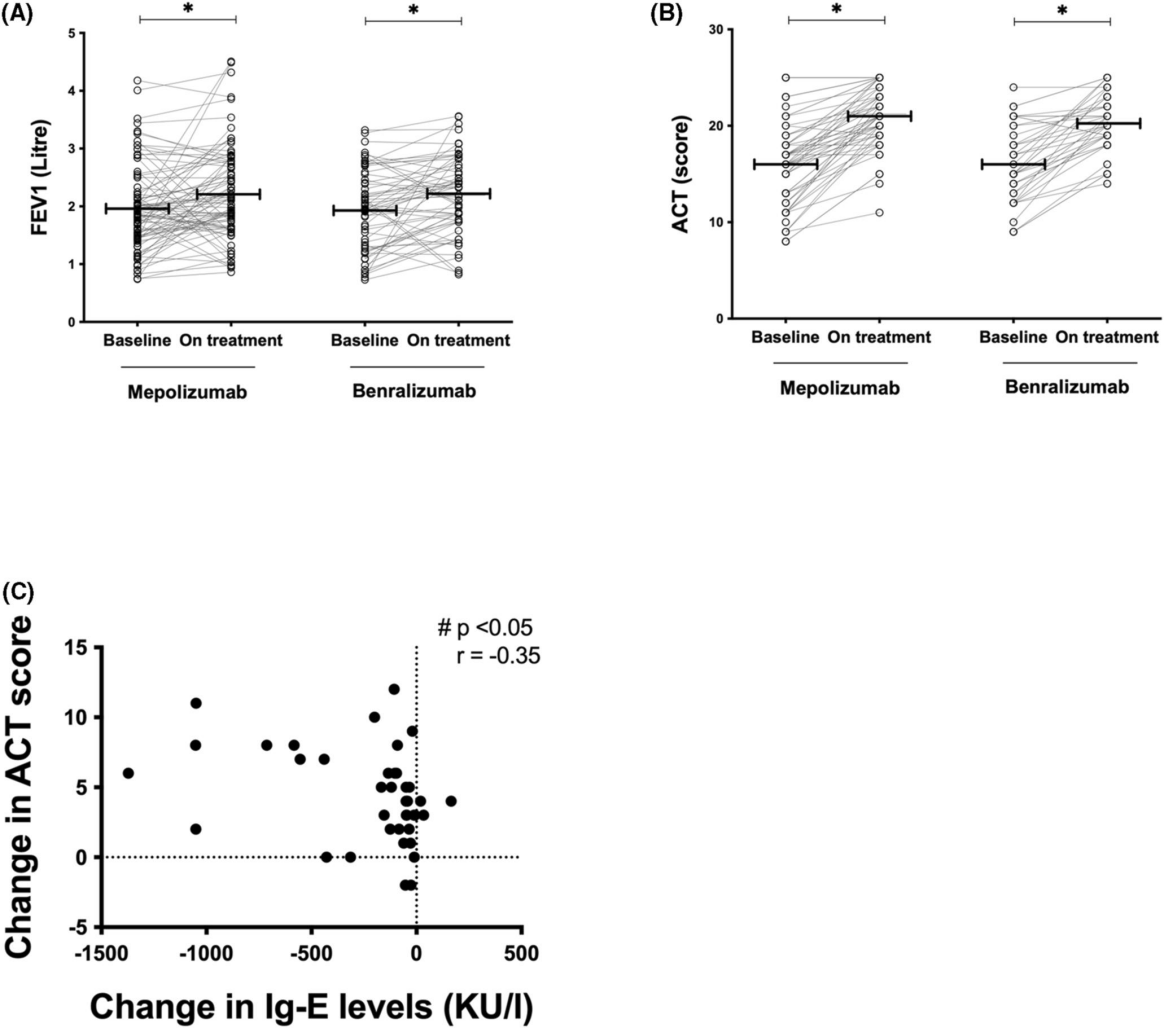
Clinical outcomes. (A) Lung function (FEV1) at baseline (before biologic treatments) and on‐treatment in severe eosinophilic asthmatic patients treated with Mepolizumab or Benralizumab. (B) Asthma control (assessed by asthma control test questionnaire [ACT]) at baseline (before biologic treatments) and on‐treatment in severe eosinophilic asthmatic patients treated with mepolizumab or benralizumab. Correlations between the on‐treatment change in ACT score and the change in total blood IgE levels (C) (# unadjusted *p*‐value for multiple testing)

Lung function assessments before and after monoclonal antibody treatments were available for 81% of patients treated with mepolizumab (*n* = 84) and 72% of patients treated with benralizumab (*n* = 59; 121 ± 4 vs. 117 ± 4 days of treatment, respectively; *p* > 0.05). Pre‐bronchodilator FEV1 significantly improved in both groups (Figure 4A). No correlations were found between changes in lung function and change in blood IgE levels. The ACT scores before and after monoclonal antibody treatment were available for 57% (*n* = 60) of patients treated with mepolizumab and for 47% (*n* = 39) of patients treated with benralizumab (121 ± 4 vs. 120 ± 5 days of treatment, respectively; *p* > 0.05). The ACT scores were clinically improved in both groups (Figure 4B). In patients treated with benralizumab, we found a weak but statistically significant negative correlation between the on‐treatment change in ACT score with change in IgE levels (*p* < 0.05; *r* = −0.32; Figure 4C). This was confirmed by Holm‐Bonferroni correction for multiple comparisons (adjusted *p*‐value 0.048).

## 4 DISCUSSION

In this retrospective, multicentre study, we found that similar durations of mepolizumab and benralizumab treatments (approximately 3 months) resulted in different effects on total blood IgE levels. While mepolizumab administration did not significantly modify the levels of total IgE in the blood during the study period, benralizumab administration led to a significant reduction in total blood IgE levels. Interestingly, and in line with recently published preliminary data,^25^ we found that benralizumab strongly reduced blood basophil counts in the study population. In patients treated with benralizumab, the data suggest a correlation between the reduction in total blood IgE levels with the reduction of blood basophils (but not eosinophils) and with the improvement of asthma control. These latter correlations require assessment in properly designed longitudinal studies.

Mepolizumab and benralizumab are effective treatments that can reduce the need for systemic corticosteroid treatment and the exacerbation rate in patients with eosinophilic severe asthma.^8^, ^9^, ^10^, ^11^, ^12^, ^13^ The clinical and immunological profiles of patients with severe asthma are rarely black and white, and there is an overlap of indications for different biological treatments in these patients.^2^, ^3^ The impact of an anti‐IL‐5‐pathway treatment on mechanisms beyond targeting eosinophils is associated with different pathways (e.g., IgE) and clinical traits (e.g., atopic status), opening up possible personalised treatment for patients with such mixed profiles.

Both mepolizumab and benralizumab inhibit eosinophilic inflammation, but the mechanisms and pathways involved are different.^6^, ^7^ Whether these differences could impact other key immune‐inflammatory elements of the T2 inflammatory cascade, such as IgE, remains largely unknown. IgE is a key regulatory molecule in T2 inflammatory response. IgE reduction can influence acute (acting mainly through interaction of IgE with mast cells and basophils) and delayed (mediated by interaction of IgE with high‐affinity IgE receptors expressed on regulatory and effector cells) allergen‐driven inflammatory responses.^14^ These events can affect clinical outcomes and interfere with other important pathways that are dysregulated in severe asthma.^26^ Notably, in our study, we found a weak but significant correlation between improvement in asthma control and a reduction in total IgE levels in patients treated with benralizumab. The observed reduction was consistent and independent of the benralizumab pharmacological regimen adopted (monthly vs. bimonthly administration), in line with data from randomised clinical trials^12^, ^27^ showing no loss of inhibitory effect or augmentation of other biomarkers (including blood eosinophil levels) after switching the benralizumab regimen to bimonthly. Recently, limited within‐patient variability was shown in total blood IgE levels over 1 year, in severe asthmatic patients (mainly atopic subjects) not treated with biologics.^28^ This natural trend was similar to the trend we observed in mepolizumab‐treated patients, while a reduction in blood IgE levels was observed in the vast majority (92%) of patients treated with benralizumab.

The mechanisms underlying the different biological behaviours in terms of the modulation of IgE levels in benralizumab versus mepolizumab treatment are unknown. Interestingly, in line with a recent publication,^25^ we found that benralizumab strongly reduced blood basophil counts in the study population, while mepolizumab showed a marginal effect. The different effects of the treatments on basophils could be due to the different mechanisms of action of the two compounds. Indeed, benralizumab, but not mepolizumab, can bind to the IL‐5 receptor highly expressed on basophils, leading to antibody‐dependent cell‐mediated cytotoxicity.^7^, ^25^ IL‐5R‐expressing cells, including basophils and eosinophils, are important sources of chemokines and cytokines, such as IL‐4 and IL‐13.^29^ IL‐4 is the most important cytokine involved in IgE regulation and induction. IL‐13 is a pleiotropic T2 cytokine that also promotes NO‐synthase activity and NO production. Thus, the stronger depletion of basophils and eosinophils induced by benralizumab compared to mepolizumab could be involved in the different effects on IgE levels as well as could contribute to the greater FeNO reduction observed with anti‐IL5‐Ra. To test this hypothesis, it would be necessary to assess the modulatory effect of benralizumab on the basophil release of mediators involved in IgE production (such as IL‐4 and IL‐13) under experimental conditions.

To the best of our knowledge, this is the first study to assess the effects of anti‐IL‐5(R) antibodies on circulating IgE levels. However, the study is not without caveats. First, we included in the analysis only atopic patients not treated with chronic oral corticosteroids (OCS). The choice was made because we aimed to include patients with clinical indications for anti‐IgE treatment and to avoid the potential effects of OCS on IgE levels,^30^ which could bias the interpretation of the results. Thus, the possible influence of chronic oral corticosteroid treatment on our findings has not been evaluated and deserves a separate study (avoiding a mixed population of OCS‐ and non‐OCS‐treated patients). Second, the study period was short (4 ± 2 months). However, the currently available literature shows that biological treatments have rapid (days) and persistent effects on the tested biomarkers^28^, ^31^, ^32^; thus, a longer period of observation should not affect the measured outcome. Third, our study was not meant to evaluate the effects of the observed interference/correlation between the two pathways on clinical outcomes. We found a weak correlation between the reduction of total blood IgE levels and the improvement of asthma control. These findings should be considered as hypothesis‐generating for a properly designed and powered perspective study, possibly assessing whether these biological differences result in clinically relevant outcomes. Finally, this is a retrospective study. Randomised and longitudinal prospective trials are needed to confirm these clinical findings. Nonetheless, since the patients were selected based on the real‐life use of these drugs in severe asthma centres, it turns out that on average, the clinical/biological characteristics of the patients included in the analyses is well balanced between the two groups, which we consider an added value in supporting the robustness of our findings.

In conclusion, in this study, we found that mepolizumab did not reduce circulating IgE levels, whereas benralizumab did. The different mechanisms of action for anti‐IL5‐pathway treatments may explain such a different impact on the relevant component of T2 inflammation in severe asthma. In this study, the effects of treatments (benralizumab vs. mepolizumb) were primarily evaluated on biological outcomes (primarily blood IgE levels but also blood inflammatory cell counts). The clinical impact of our findings should be assessed in properly designed studies.

## CONFLICT OF INTEREST

Marco Contoli reports grants, personal fees and non‐financial support from Chiesi, personal fees and non‐financial support from AstraZeneca, personal fees and non‐financial support from Boehringer Ingelheim, personal fees and non‐financial support from Alk‐Abello, grants, personal fees and non‐financial support from GlaxoSmithKline, personal fees and non‐financial support from Novartis, personal fees and non‐financial support from Zambon, grants from University of Ferrara, Italy, outside the submitted work. Chiara Martelli reports personal fees and non‐financial support from GSK, Astrazeneca, Novartis, Sanofi outside the submitted work. Alberto Papi reports grants, personal fees, non‐financial support and other from GlaxoSmithKline, grants, personal fees and non‐financial support from AstraZeneca, grants, personal fees, non‐financial support and other from Boehringer Ingelheim, grants, personal fees, non‐financial support and other from Chiesi Farmaceutici, grants, personal fees, non‐financial support and other from TEVA, personal fees, non‐financial support and other from Mundipharma, personal fees, non‐financial support and other from Zambon, personal fees, non‐financial support and other from Novartis, grants, personal fees and non‐financial support from Menarini, personal fees, non‐financial support and other from Sanofi/Regeneron, personal fees from Roche, grants from Fondazione Maugeri, grants from Fondazione Chiesi, personal fees from Edmondpharma, outside the submitted work. The remaining authors declare that the research was conducted in the absence of any commercial or financial relationships that could be construed as a potential conflict of interest.

## AUTHOR CONTRIBUTIONS

**Marco Contoli:** Conceptualization; Data curation; Formal analysis; Investigation; Methodology; Project administration; Supervision; Validation; Writing — original draft. **Pierachille Santus:** Data curation; Formal analysis; Investigation; Methodology; Validation; Writing — review & editing. **Francesco Menzella:** Data curation; Formal analysis; Investigation; Validation; Writing — review & editing. **Cindy Rocchi:** Data curation; Investigation; Supervision; Writing — review & editing. **Dejan Radavanovic:** Data curation; Formal analysis; Investigation; Validation; Writing — review & editing. **Federico Baraldi:** Data curation; Investigation; Writing — review & editing. **Chiara Martelli:** Data curation; Investigation; Writing — review & editing. **Serena Casanova:** Data curation; Investigation; Writing — review & editing. **Carlo Barbetta:** Data curation; Formal analysis; Investigation; Validation; Writing — review & editing. **Claudio Micheletto:** Data curation; Formal analysis; Investigation; Supervision. **Nicola Scichilone:** Data curation; Formal analysis; Investigation; Methodology; Supervision. **Bianca Beghe:** Data curation; Investigation; Supervision. **Elisiana Carpagnano:** Data curation; Formal analysis; Investigation; Supervision. **Alberto Papi:** Data curation; Formal analysis; Methodology; Validation; Writing — original draft.

## Supporting information

Supporting Information S1

Supporting Information S2
