# Supplementary material for: Effects of anti‐IL5 biological treatments on blood IgE levels in severe asthmatic patients: A real‐life multicentre study (BIONIGE)
Source: Clin Transl Allergy. 2022 Apr 7;12(4):e12143. doi: 10.1002/clt2.12143 (PMC8988861; doi:10.1002/clt2.12143)
Supplement: Supplementary file 1 — Supporting Information S1 [file CLT2-12-e12143-s001.docx]

**Supporting Information**

**Effects of anti-IL5 biological treatments on blood IgE levels in severe asthmatic patients: a real-life multicentre study (BIONIGE)**

Marco Contoli^1^*, PhD, MD; Pierachille Santus^2^, PhD, MD; Francesco Menzella^3^, MD; Cindy Rocchi^1^, MD; Dejan Radovanovic^2^, MD; Federico Baraldi^1^, MD; Chiara Martelli^1^, MD; Serena Casanova^1^, MD; Carlo Barbetta^4^, MD; Claudio Micheletto^5^, MD; Nicola Scichilone^6^ PhD, MD; Bianca Beghè^7^, MD, Elisiana Carpagnano^8^, MD, Alberto Papi^1^, MD.

^1^ Respiratory Medicine, Department of Translational Medicine, University of Ferrara, Ferrara, Italy and Emergency Department, University Hospital S. Anna, Ferrara, Italy

^2^ Division of Respiratory Diseases, Ospedale Luigi Sacco, Polo Universitario, ASST Fatebenefratelli-Sacco, Department of Biomedical and Clinical Sciences (DIBIC), Università Degli Studi di Milano, Milan, Italy.

^3^ Pneumology Unit, Arcispedale Santa Maria Nuova, Azienda USL-IRCCS di Reggio Emilia, Reggio Emilia, Italy

^4^ Department of Pulmonary Medicine, Ospedale Santa Maria degli Angeli, Pordenone, Italy.

^5^ Cardio-Thoracic Department, Respiratory Unit, Integrated University Hospital, Verona, Italy

^6^ Dipartimento Universitario di Promozione Della Salute, Materno Infantile, Medicina Interna e Specialistica di Eccellenza "G. D'Alessandro" (PROMISE), Division of Respiratory Medicine, "Paolo Giaccone" University Hospital, University of Palermo; Palermo; Italy.

^7^ Respiratory Diseases Unit, Department of Medical and Surgical Sciences, University of Modena and Reggio Emilia, Italy.

^8^ Division of Respiratory Diseases, Department of Medical and Surgical Sciences, University of Foggia, Italy & Cardiothoracic Dept, Respiratory and Critical Care Unit, Polyclinic University Hospital, Bari, Italy

**Corresponding Author Information.**

*Marco Contoli, MD, PhD

Associate professor Respiratory Medicine

Department of Translational Medicine

University of Ferrara (Italy)

Via Ariosto 35, 44121 Ferrara Italy

Phone: +390532688148

Email: [ctm@unife.it](mailto:ctm@unife.it)

**Study population**

21 patients (20%) in the mepolizumab treated group and 14 patients (17%) in the Benralizumab treated group were sensitized to seasonal allergens only.

Despite a different threshold of blood eosinophils for mepolizumab and benralizumab prescription, we found that only 15 patients (14%) in the mepolizumab group showed at baseline blood eosinophil levels ranging from 150 to 300 cells/µl.

All patients were on high dose inhaled corticosteroids/long acting β2 bronchodilators (ICS/LABA) fixed combinations (Step 5 GINA) ^1^. Thirty-two (31%) patients in the mepolizumab group and 27 (33%) patients in the benralizumab group, were treated also with inhaled long-acting muscarinic receptor antagonists (LAMA).

Standardized differences of means (for continuous variables) and prevalence (for dichotomous variables) of baseline demographic, clinical and biological characteristics of the two groups of patients we calculated. Standardised differences between groups were laid within a 10% window (standardised difference <0.1) that met the criterion of a negligible difference ^2,3^ (Table S1). These data confirm that the two population of patients included in the real-life retrospective analysis comparable at baseline.

**IgE levels**

Considering the different regimens of administration of the two treatments after the initial 3 monthly doses (i.e. monthly vs. bimonthly administration for mepolizumab and benralizumab, respectively) we evaluated the blood IgE levels in patients with the assessment of blood total IgE performed 4-6 months after the initiation of biological treatments (i.e. in patients still on monthly prescription of mepolizumab but already bimonthly prescription of Benralizumab). The result was consistent with the main finding. No significant reduction compared to baseline was found in total blood IgE levels in patients treated with mepolizumab and a significant reduction (approximately 36% reduction) of IgE levels was found in patients treated with Benralizumab (401±63 vs 397±77 kU/I, in mepolizumab treated patients (n=45); p>0.05; 409±83 vs 270±53 kU/I, in benralizumab treated patients (n=29); p<0.05). This data shows that the blood IgE reduction was consistent and independent of the benralizumab pharmacological regimen adopted (monthly vs. bimonthly administration), in line with data from randomised clinical trials ^4,5^ showing no loss of inhibitory effect or augmentation of other biomarkers (including blood eosinophil levels) after switching the benralizumab regimen to bimonthly. The effect of treatments on blood IgE levels was also evaluated with the exclusion, in the mepolizumab treated group, of patients with low blood eosinophils (< 300 cells/mcl – n=15). Consistently, we found that mepolizumab administration did not modify IgE values even when patients with low blood eosinophil levels (n=15) were excluded (405±53 and 411±55 kU/I at baseline and after treatment with mepolizumab, respectively).

To exclude an acute/transient effect of the administration of biological treatments on blood IgE levels, we calculate in the two groups the days of IgE assessment since last administration of treatments. We found a longer period of time since last administration of biological treatments to IgE assessment in patients treated with Benralizumab compared to Mepolizumab (30±2 vs 14±1 days; p<0.001). These data support a chronic rather than acute effect of Benralizumab on the evaluated outcome.

An additional analysis including patients with all the demographic, clinical, functional and biological characteristics at baseline available [88 patients (85%) and 66 (80%) of Mepolizumab and Benralizumab treated patients, respectively], was performed. This analysis provided similar results to those originally presented when considered the entire population. Mepolizumab did not modified blood IgE values (412±52 vs 415±53 kU/I), while Treatment with Benralizumab was associated with a significant reduction of total IgE compared to baseline (412±92 kU/l vs 254±42, -39%, p<0.001).

**Safety of treatments**

Side effects were collected as part of the clinical management of patients receiving biological treatments. Only three minor side effects were reported: an episode of self-limiting abdominal pain that lasted for approximately 6 hours the day after mepolizumab administration in a 45 year old man; an exacerbation of migraine that did not required medical intervention the same day of benralizumab administration in a 49 year old female with of chronic migraine; a transient and self-limiting pain in the site of mepolizumab subcutaneous injection that occurred the day after the administration. In line with previous randomised controlled trials ^4,6–10^ , no major side effects have been documented

**References**

1. Global Initiative for Asthma. Global Strategy for Asthma Management and Prevention, 2021. Available from: www.ginasthma.org. Published online April 27, 2021:1-217.

2. Austin PC. An Introduction to Propensity Score Methods for Reducing the Effects of Confounding in Observational Studies. Multivar Behav Res. 2011;46(3):399-424. doi:10.1080/00273171.2011.568786

3. Austin PC. Balance diagnostics for comparing the distribution of baseline covariates between treatment groups in propensity-score matched samples. Stat Med. 2009;28(25):3083-3107. doi:10.1002/sim.3697

4. Bleecker ER, Fitzgerald JM, Chanez P, et al. Efficacy and safety of benralizumab for patients with severe asthma uncontrolled with high-dosage inhaled corticosteroids and long-acting β2-agonists (SIROCCO): a randomised, multicentre, placebo-controlled phase 3 trial. Lancet. 2016;388(10056):2115-2127. doi:10.1016/s0140-6736(16)31324-1

5. Busse WW, Bleecker ER, Fitzgerald JM, et al. Long-term safety and efficacy of benralizumab in patients with severe, uncontrolled asthma: 1-year results from the BORA phase 3 extension trial. The Lancet Respiratory Medicine. 2019;7(1):46-59. doi:10.1016/s2213-2600(18)30406-5

6. Pavord ID, Korn S, Howarth P, et al. Mepolizumab for severe eosinophilic asthma (DREAM): a multicentre, double-blind, placebo-controlled trial. Lancet. 2012;380(9842):651-659. doi:10.1016/s0140-6736(12)60988-x

7. Bel EH, Wenzel SE, Thompson PJ, et al. Oral Glucocorticoid-Sparing Effect of Mepolizumab in Eosinophilic Asthma. N Engl J Med. 2014;371(13):140908054511000. doi:10.1056/nejmoa1403291

8. Ortega HG, Liu MC, Pavord ID, et al. Mepolizumab Treatment in Patients with Severe Eosinophilic Asthma. N Engl J Med. 2014;371(13):140908054511000. doi:10.1056/nejmoa1403290

9. Castro M, Wenzel SE, Bleecker ER, et al. Benralizumab, an anti-interleukin 5 receptor α monoclonal antibody, versus placebo for uncontrolled eosinophilic asthma: a phase 2b randomised dose-ranging study. The Lancet Respiratory Medicine. 2014;2(11):879-890. doi:10.1016/s2213-2600(14)70201-2

10. Nair P, Wenzel S, Rabe KF, et al. Oral Glucocorticoid-Sparing Effect of Benralizumab in Severe Asthma. N Engl J Med. 2017;376(25):2448-2458. doi:10.1056/nejmoa1703501

**Table S1.** The standardised difference (d) between means (for continuous variables) and between prevalence (for dichotomous variables) was calculated to compare the baseline characteristics of the mepolizumab- and benralizumab-treated groups.

|  | **d**  **Standardized difference** |
| --- | --- |
| **Age** | -0,0583 |
| **Gender** | -0,0032 |
| **Smoking habit (pack/year)** | 0,0515 |
| **Lung function (FEV1 litre)** | 0,0135 |
| **ACT score** | -0,0401 |
| **Comorbid conditions** |  |
| Obesity BMI>30 | -0,0921 |
| Atopic dermatitis | -0,050 |
| Chronic rhinosinusitis including nasal polyps, | -0,0018 |
| Gastroesophageal reflux disease | -0,0053 |
| Anxiety-depression syndrome | -0,0150 |
| **Blood total IgE, kU/l** | -0,0294 |
| **Blood Eosinophils** | -0,0773 |
