# Supplementary material for: Effects of anti‐IL5 biological treatments on blood IgE levels in severe asthmatic patients: A real‐life multicentre study (BIONIGE)
Source: Clin Transl Allergy. 2022 Apr 7;12(4):e12143. doi: 10.1002/clt2.12143 (PMC8988861; doi:10.1002/clt2.12143)
Supplement: Supplementary file 2 — Supporting Information S2 [file CLT2-12-e12143-s002.docx]

**Appendix 1. Recruitng centres.**

| Local PI | Centre |
| --- | --- |
| Prof. Marco Contoli | UO Pneumologia - Azienda Ospedaliero Universitaria di Ferrara. Italy |
| Prof. Pierachille Santus | UOC Pneumologia Ospedale L.Sacco – Polo Univestiario ASST Fatebenefratelli – Milano. Italy |
| Dr. Claudio Micheletto | UOC Pneumologia Azienda Ospedaliera Univeristaria Integrata – Verona - Italy |
| Dr. Francesco Menzella | Department of Medical Specialties, Pneumology Unit, Arcispedale Santa Maria Nuova, Azienda USL di Reggio Emilia-IRCCS |
| Dr. Carlo Barbetta | Department of Pulmonary Medicine, Ospedale Santa Maria degli Angeli, Pordenone, Italy |
| Prof. Nicola Scichilone | Division of Respiratory Medicine, "Paolo Giaccone" University Hospital, University of Palermo; Palermo; Italy. |
| Prof.ssa Bianca Beghè | Respiratory Diseases Unit, Department of Medical and Surgical Sciences, University of Modena and Reggio Emilia, Italy |
| Prof. Elisiana Carpagnano | Division of Respiratory Diseases, Department of Medical and Surgical Sciences, University of Foggia, Italy |
